# New Carbon Allotropes with Helical Chains of Complementary Chirality Connected by Ethene-type *π*-Conjugation

**DOI:** 10.1038/srep03077

**Published:** 2013-10-29

**Authors:** Jian-Tao Wang, Changfeng Chen, Yoshiyuki Kawazoe

**Affiliations:** 1 Beijing National Laboratory for Condensed Matter Physics, Institute of Physics, Chinese Academy of Sciences, Beijing 100190, China; 2 Department of Physics and High Pressure Science and Engineering Center, University of Nevada, Las Vegas, Nevada 89154, USA; 3 New Industry Creation Hatchery Center, Tohoku University, Sendai, 980-8579, Japan; 4 Institute of Thermophysics Siberian Branch, Russian Academy of Science, Novosibirsk, 630090, Russia

## Abstract

We here identify by *ab initio* calculations two distinct three-dimensional three-connected (3D3C) chiral framework structures of carbon in 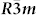 and *I*4_1_/*amd* symmetry, respectively, which comprise 3-fold and 4-fold helical chains with complementary chirality. The helical carbon chains are connected by an ethene-type planar *π*-conjugation, and the resulting structures contain a network of *sp*^2^ carbon bonds with one-third being double bonds between the chains and two-thirds single bonds along the chains. Phonon and electronic band structure calculations show that these chiral carbene structures are dynamically stable and exhibit a large band gap (2.4 ~ 2.9 eV). This semiconducting nature reflects a key distinction from previously proposed metallic isomers of helical or zigzag carbon chains with twisted *π* states that are dynamically unstable. The present results solve the long-sought 3D3C all-*sp*^2^ carbon structures and may help design other covalent bonding networks.

Carbon exhibits an extremely rich variety of allotropic forms^1^ that possess a wide range of properties with numerous applications in many areas of science and technology. Despite the existence of a large number of known carbon allotropes, the quest for new carbon structures has been a very active research field. The valence electrons of the carbon atom are capable of forming *sp*^3^-, *sp*^2^- and *sp*-hybridized states that support four basic types of single, double, triple, and aromatic carbon-carbon bonds, which are closely related to the bonding configurations in ethane, ethene, ethyne, and benzene-type hydrocarbon structures (see Fig. 1 for a comparative illustration). At ambient conditions, graphite, which is structurally related to polycyclic benzenoid aromatic hydrocarbon, is the most thermodynamically stable carbon configuration (see Fig. 2). The polycyclic carbon atoms form a two-dimensional three-connected (2D3C) benzenoid *sp*^2^ bonding network with bond angles of 120° and bond lengths of 0.142 nm. Diamond, which is related to polycyclic saturated hydrocarbon, is the second most stable allotrope of carbon with all the carbon atoms in a methane-like tetrahedral *sp*^3^ bonding with bond angles of 109.5° and bond lengths of 0.154 nm as in alkanes, forming a very rigid 3D4C carbon network. Interestingly, the simplest 1D2C *sp*-carbyne has a polyyne-like alternating single and triple carbon-carbon bonds rather than a cumulene structure^2^ and it has been recently synthesized^3^ despite its rather high energy of ~1 eV per atom above that of graphite.

Beside carbyne, graphite and diamond, several other forms of carbon also have been synthesized; these include 0D3C fullerenes^4^, 1D3C nanotubes^5^, 2D3C graphene^6^, and 3D4C cold-compressed graphite^7^,^8^,^9^,^10^,^11^,^12^,^13^ and fullerene^14^. Conspicuously missing from this list of carbon structures is the long-sought 3D3C carbon in all-*sp*^2^ networks. The first 3D3C carbon structure, the so-called polybenzene (PBz)^15^,^16^,^17^ was proposed in 1946; it contains two intertwined polyphenylene helices^18^. Various other hypothetical carbon modifications^19^ in all 3D3C network have also been proposed, including the metallic hexagonal H-6 carbon^20^, ThSi_2_-type tetragonal bct-4 carbon^21^, and SrSi_2_-type cubic *K*_4_ carbon^22^. It has been shown, however, these hypothesized structures are dynamically unstable^23^. Finding stable 3D3C carbon framework structures remain hitherto an elusive goal that continues to attract considerable interest. A crucial task here is to understand the underlying mechanism responsible for the instability of previously proposed 3D3C carbon structures and, consequently, design new structures that avoid such pitfalls.

Here, we present the results of *ab initio* total-energy and phonon calculations^24^,^25^,^26^,^27^,^28^,^29^ that identify two three-dimensional carbon structures with all-*sp*^2^ bonding that are dynamically stable and energetically metastable. These newly identified 3D3C allotropic forms of carbon comprise helical chains with complementary chirality connected by ethene-type (H_2_C = CH_2_) *π*-conjugation, thus termed chiral carbene. One structure has an 18-atom hexagonal unit cell in 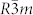 (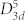) symmetry with six 3-fold helical chains, which topologically corresponds to the 2D star lattice^30^; and the other structure has a 16-atom body-centered tetragonal unit cell in *I*4_1_/*amd* (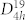) symmetry with four 4-fold helical chains, which topologically corresponds to the 2D square lattice^31^. The bonding configuration and distribution in chiral carbene are distinct from those in carbyne, graphite and diamond, one-third of the bonds are double bonds between the chains and two-thirds are single bonds along the chains. Electronic band structure calculations show that chiral carbene exhibits semiconducting character with a considerable band gap of 2.4 ~ 2.9 eV. This semiconducting nature reflects the favorable underlying bonding configuration in chiral carbene, which is its key distinction from previously proposed isomers that contain helical chains of same chirality (e.g., *K*_4_) or zigzag chains (e.g., H-6 and bct-4), all of which have unmatched torsion angles that would reduce or cut the *π*-conjugation and produce metallic states in these structures. The complementary helical chain arrangement in chiral carbene allows effective strain release, thus enhancing the structural stability. This new design principle may also offer insights for constructing and understanding other covalent bonding networks.

## Results

**Figure 1:**
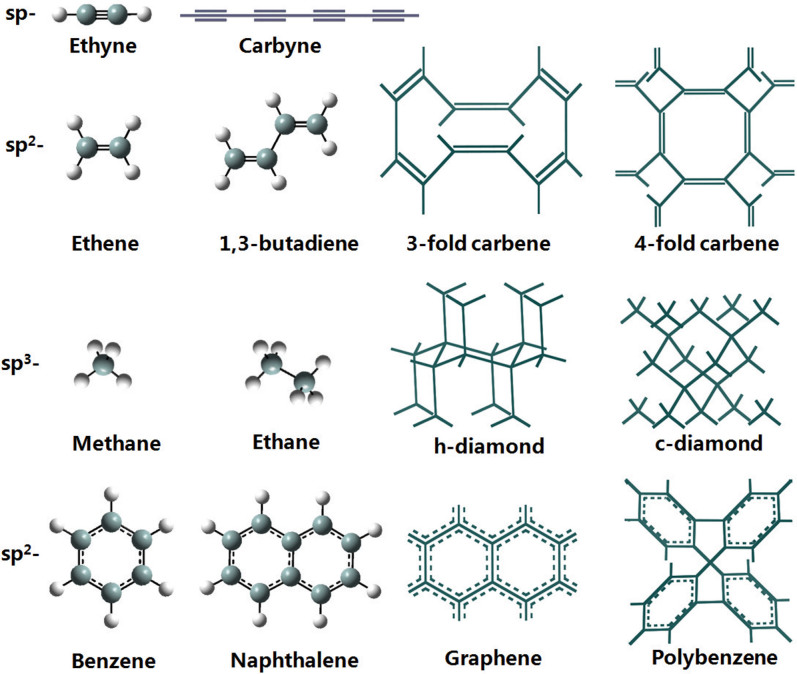
Carbon-carbon bonds in *sp*-, *sp*^2^- and *sp*^3^-hybridized states. A comparative illustration of basic bonding configurations in ethyne, ethene, ethane, and benzene-type hydrocarbons and those in pure carbon allotropes, among which the 3-fold and 4-fold carbene are newly identified in this work. The typical carbon-carbon bond lengths with distinct bonding configurations are 1.54 Å for C(*sp*^3^)–C(*sp*^3^) single bonds as in diamond and ethane; 1.46 Å for C(*sp*^2^)–C(*sp*^2^) single bonds as in 1,3-butadiene and 1.48 Å in 3-fold and 4-fold carbene; 1.40 Å for aromatic bonds as in benzene and 1.42 Å in graphite; 1.34 Å for C(*sp*^2^) = C(*sp*^2^) double bonds as in ethene, 1,3-butadiene, 3-fold and 4-fold carbene; and 1.20 Å for C(*sp*)≡C(*sp*) triple bonds as in ethyne and carbyne.

We first present the results on the structural configuration of the 3-fold chiral crystalline form of carbon. It has a 6-atom rhombohedral primitive unit cell (termed cR6 carbon or 3-fold carbene) in 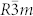 (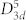) symmetry with lattice parameters *a* = 4.2442 Å and *γ* = 112.415°, occupying the 6h (0.5952, 0.000, 0.4048) position. In hexagonal representation, as shown in Fig. 3a, it has an 18-atom unit cell with equilibrium lattice parameters *a* = 7.0544 Å and *c* = 3.5817 Å, occupying the 18f (0.4054, 0.000, 0.000) position, comprising three left-handed (S) and three right-handed (R) helical chains, topologically corresponding to the 2D star lattice^30^. It is shown as an (8,3)-net structure. Each carbon chain has three neighboring chains of complementary chirality with 3-fold screw axes running parallel to the z-axis. The six helical chains bond together forming a closed C_12_ armchair ring with alternating single and double carbon-carbon bonds, and two neighboring helical chains form a closed C_8_ zigzag ring with two double bonds as in 1,5-cyclooctadiene. Thus, it contains two distinct bond lengths, a longer bond of 1.479 Å associated with the C(*sp*^2^)-C(*sp*^2^) single bond along the chains and a shorter bond of 1.344 Å associated with the C(*sp*^2^) = C(*sp*^2^) double bond between the chains. There are also two different bond angles, 118.52° for ∠C-C-C along the chain and 120.74° for ∠C-C = C out of the chain. These bonding parameters on average are similar to those in graphite, but are closer to those in ethene and 1,3-butadiene molecules (see Fig. 1), thus cR6 is a 3-fold chiral carbene.

**Figure 2:**
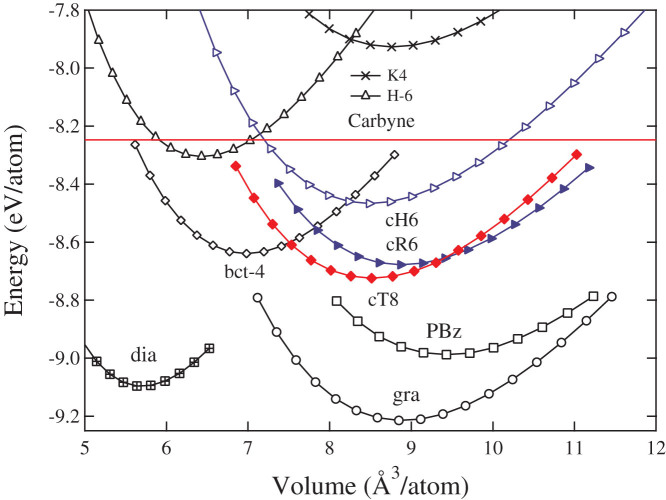
Energy versus volume per carbon atom for various carbon structures under GGA method. *K*_4_, H-6, bct-4, cH6, cR6, cT8 and PBz in 3D3C bonding networks are plotted in comparison with 1D2C carbyne, 2D3C graphite and 3D4C diamond.

**Figure 3:**
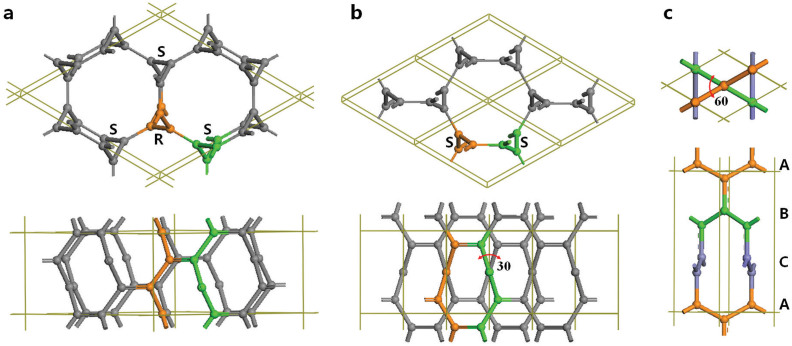
Top (upper panel) and side (lower panel) views of the the 3-fold chiral crystalline structures. (a) cR6 in 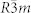 (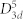) symmetry containing three left-handed (S) and three right-handed (R) chains. (b) cH6 in *P*6_2_22 (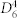) symmetry containing two left-handed (S) chains with unmatched torsion angles of 30° between the helical chains. (c) H-6 in *P*6_2_22 (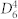) symmetry^20^ containing three planar zigzag chains in ABC stacking with unmatched torsion angles of 60° between the zigzag chains.

The matching complementary chirality of the neighboring chains in the cR6 carbon phase is crucial to its structural stability. To illustrate this point, we construct a similar 3-fold chiral crystalline structure that comprises 3-fold left-handed (S) helical chains in *P*6_2_22 (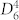) symmetry as shown in Fig. 3b. It has a 6-atom hexagonal unit cell (named cH6 hereafter) with lattice parameters *a* = 3.8867 Å, and *c* = 3.7591 Å, occupying the 6h (0.3975, 0.6025, 0.3333) position. Each chain in cH6 structure is surrounded by three chains of the same chirality with 3-fold screw axes running parallel to the z-axis. Six chains form a twist C_14_ ring and two chains bond together tend to form a twisted C_8_ ring with an unmatched torsion angle of 30° (Fig. 3b), which weakens the double bond by elongating the bond length to 1.39 Å from 1.34 Å and increases the total energy of cH6 relative to that of cR6 (see Fig. 2). The previously reported H-6 carbon^19^, a (10,3)-net structure, has the same *P*6_2_22 symmetry as in cH6, and it comprises three infinite planar zigzag chains in ABC stacking as shown in Fig. 3c. This structure contains a large unmatched torsion angle of 60° between the zigzag chains, which leads to an elongated bond length of 1.45 Å between the zigzag chains, and thus induces energetic instability (see Fig. 2). These results show that the isomers comprising 3-fold helical chains with the same chirality or planar zigzag chains with a torsion angle (twisted *π*-bonding) are less favorable than the chiral structure comprising helical chains of complementary chirality connected by ethene-type planar *π*-bonding.

**Figure 4:**
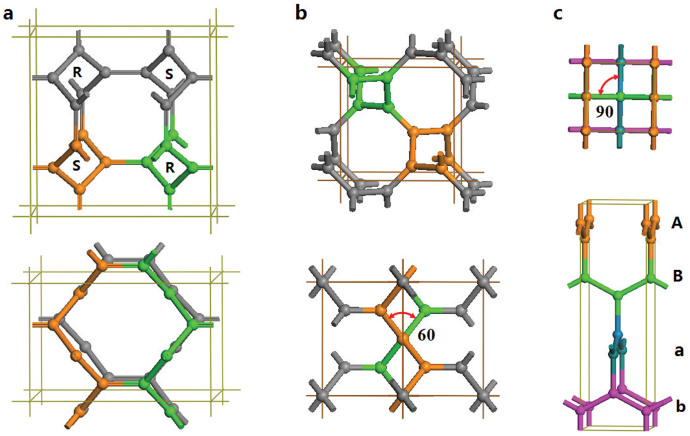
Top (upper panel) and side (lower panel) views of the 4-fold chiral crystalline structures. (a) cT8 in *I*4_1_/*amd* (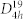) symmetry containing two left-handed (S) and two right-handed (R) chains. (b) *K*_4_ in *I*4_1_32 (*O*^8^) symmetry^22^ containing two right-handed chains with unmatched torsion angles of 60° between the helical chains. (c) bct-4 in *I*4_1_/*amd* (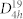) symmetry^21^ containing four planar zigzag chains in ABab stacking with unmatched torsion angles of 90° between the zigzag chains.

The second chiral crystalline form of carbon identified in this work comprises 4-fold chains with complementary chirality in *I*4_1_/*amd* (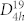) symmetry. In a conventional cell, it has a 16-atom body-centered tetragonal unit cell with equilibrium lattice parameters *a* = 5.8963 Å, and *c* = 3.7843 Å, occupying the 16f (0.25, 0.1139, 0.875) position as shown in Fig. 4a. It topologically corresponds to the 2D square lattice^31^. In the primitive unit cell, it has eight atoms (termed cT8 carbon or 4-fold carbene) with lattice parameters a = 4.5786 Å, *α* = 99.83°, *γ* = 131.18°. It is also shown as an (8,3)-net structure. Each chain in cT8 carbon has four neighboring chains of opposite chirality with 4-fold screw axes running parallel to the z-axis. Four 4-fold chains form a closed C_8_ armchair ring with alternating single and double carbon-carbon bonds as in tub-shaped 1,3,5,7-cyclooctatetraene, and two neighboring chains of opposite chirality bond together forming a closed C_10_ zigzag ring with two ethene-type planar *π*-conjugation. Similar to the situation in cR6, there are two distinct bond lengths here: a longer bond of 1.477 Å associated with the C(*sp*^2^)-C(*sp*^2^) single bond along the chains and a shorter bond of 1.343 Å associated with the C(*sp*^2^) = C(*sp*^2^) double bond between the chains. Meanwhile, there are two different bond angles of 114.70° and 122.65° along and out of the chains, respectively. These bonding data on average are similar to those in graphite, and close to those in ethene and 1,3-butadiene molecules (see Fig. 1), thus cT8 carbon is a 4-fold chiral carbene.

We now examine the previously proposed *K*_4_ and bct-4 carbon to identify the structural features that are unfavorable for their stability. The cubic *K*_4_ carbon^22^ in *I*4_1_32 (*O*^8^) symmetry (Fig. 4b) has an eight-atoms unit cell comprising two 4-fold helices with the same chirality running parallel with the three cubic axes. There is only one type of carbon-carbon bond with a bond length of 1.463 Å [close to 1.48 Å for C(*sp*^2^)-C(*sp*^2^) single bond]. Meanwhile, there is a large unmatched torsion angle of 60° between the chains that eliminates all the *π*-conjugation, yielding an unstable structure with the highest energy among the 3D3C carbon nets (see Fig. 2). The metallic bct-4 carbon^21^ has the same *I*4_1_/*amd* symmetry as that of cT8 carbon, and it comprises four infinite planar zigzag chains in ABab stacking as shown in Fig. 4c. There is a large unmatched torsion angle of 90° between the zigzag chains that cut the *π*-conjugation with an elongated bond length of 1.477 Å, leading to its less favorable energetic state compared to cT8 and graphite (see Fig. 2). Note that *K*_4_ and bct-4 carbon share a similar (10,3)-net strcuture^32^ with a four-atom primitive unit cell; *K*_4_ can be converted into bct-4 with a lattice distortion^33^; furthermore, bct-4 can be converted into a diamond-like network by contraction of the links between the zigzag chains as discussed by Batten and Robson^32^. These results suggest again that the 3D3C carbon networks comprising neighboring helices with complementary chirality are more favorable than those containing the same helical or zigzag chains.

As shown in Fig. 2, both cR6 and cT8 chiral carbon allotropes are thermodynamically metastable compared to graphite, diamond and PBz, but more stable than the metallic *K*_4_, H-6, and bct-4 carbon. The differences in energy reflect the amount of misaligned 2*p_z_* orbitals in the three-connected net structure^19^. Graphite has all its 2*p_z_* orbitals perfectly aligned, leading to a strong *π* bonding interaction between neighboring carbon atoms, producing the most stable carbon crystal. Conversely, all the 2*p_z_* orbitals are misaligned in the *K*_4_ carbon, making it the most unfavorable crystalline carbon form. Meanwhile, cR6 and cT8 have only one-third of their bonds with well-aligned 2*p_z_* orbitals via ethene-type bonding, making them less stable than PBz carbon, which has two-thirds of its bonds with well-aligned 2*p_z_* orbitals in benzene rings. It is interesting to note that although H-6 and bct-4 have two-thirds of their bonds with well-aligned 2*p_z_* orbitals along the zigzag chains, they are energetically less favorable than cR6 and cT8 because of the misalignment of the angles (60° in H-6 and 90° in bct-4) between the zigzag chains. The calculated equilibrium structural parameters, total energies, and bulk modulus for cH6, cR6 and cT8 under LDA and GGA are listed in Table I; results for diamond and graphite are also presented and compared with available experimental data^34^.

**Figure 5:**
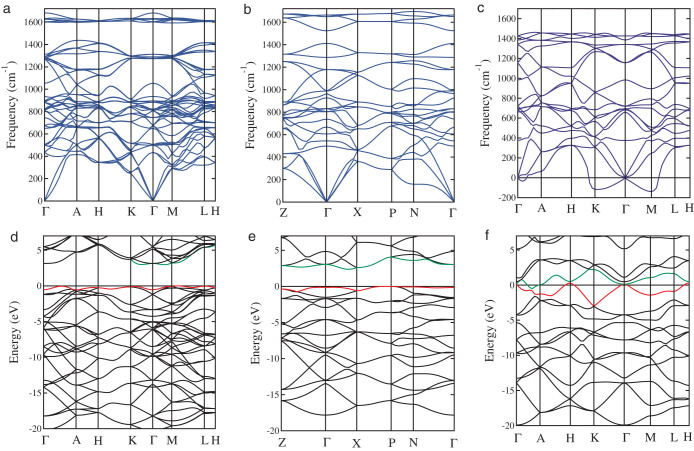
Phonon and electronic band structures. (a–c) Phonon band structures for cR6, cT8, and cH6 carbon. (d–f) Electronic band structures for cR6, cT8, and cH6 carbon. The electronic band structures were calculated using the HSE06-PBE potential.

Since energetic calculations alone cannot establish the stability of a crystal structure^23^, phonon dispersion curves were calculated for both cR6 and cT8 carbon. The calculated results shown in Fig. 5a and 5b indicate that the highest phonon frequencies are 1682 cm^−1^ for cR6 carbon and 1694 cm^−1^ for cT8 carbon, respectively, which are close to the highest phonon frequency of 1610 cm^−1^ for graphite^35^. Throughout the entire Brillioun zone, no imaginary frequencies were observed, confirming dynamical stability of the new 3-fold cR6 and 4-fold cT8 chiral crystalline allotropes of carbon in the 3D3C all-*sp*^2^-bonding networks. For comparison, the phonon dispersion curves of cH6 carbon are also plotted in Fig. 5c, and in this case, there is a visible imaginary band along the K-Γ-M direction, indicating the dynamical instability of cH6 carbon, which is caused by the twisted *π* states between the helical chains as discussed above.

Finally, we discuss the electronic properties of the 3D3C all-*sp*^2^ carbon allotropes. The electronic band structures were calculated using the hybrid functional (HSE06)^28^. As observed in PBz^16^,^17^, both cR6 and cT8 carbene exhibit semiconducting character. The cR6 carbon has an indirect band gap of 2.93 eV (Fig. 5d), and its valence band top is located along the M-L direction and the conduction band bottom is located along the K-Γ-M direction. The cT8 carbon has an indirect band gap of 2.44 eV (Fig. 5e), and its valence band top is located along the P-X direction and the conduction band bottom is located along the X-Γ direction in the Brillioun zone. For comparison, the electronic band structures of cH6 are also shown in Fig. 5f. The twisted *π* states across the Fermi level result in metallic properties of the cH6 carbon, which is similar to the situation in *K*_4_^22^, H-6^20^, and bct-4^21^ carbon. These metallic characters are directly associated with the energetically unfavorable bonding configurations that involve either elongated bond lengths or twisted bond angles. This analysis suggests that such metallic all-*sp*^2^ structures are unlikely to realize, in contrast to the chiral carbene structures proposed in the present work contain planar *π*-conjugated bonding that produces a large band-gap semiconducting state.

Recently, a one-step, gas-phase, catalyst-free detonation of hydrocarbon (C_2_H_2_) method was developed to produce gram quantities of pristine graphene nanosheets^36^. The detonation of C_2_H_2_ was carried out in the presence of O_2_. It suggests that a possible way to synthesize the new forms of carbon reported in the present work is thermal decomposition or detonation of carbonaceous materials like ethyne, ethene, 1,3-butadiene, 1,5-cyclooctadiene, and tub-shaped 1,3,5,7-cyclooctatetraene molecules. It is also worth noting that although cR6 and cT8 carbon are thermodynamically metastable compared to graphite and diamond, they are kinetically stable at ambient conditions because of the high (~0.5 eV) reaction barriers, which are calculated using a generalized solid-state nudged elastic band method^9^,^37^,^38^.

## Discussion

In summary, we have identified by *ab initio* calculations two new 3D3C chiral framework structures in all-*sp*^2^ bonding networks. The first is a 3-fold chiral carbene with an 18-atom unit cell in 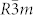 (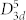) symmetry, and the second is a 4-fold chiral carbene with a 16-atom unit cell in *I*4_1_/*amd* (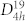) symmetry. These structures contain only one kind of *sp*^2^-bonded carbon atoms connected by ethene-type planar *π*-conjugation between the chiral chains; they are dynamically stable, thermodynamically metastable compared to graphite and are semiconducting with a large indirect band gap. Our work identifies that structures with matching helical chains of complementary chirality with ethene-type planar *π*-conjugation are energetically more favorable than structures with helical chains of same chirality or zigzag chains with twisted *π* bonds. The present results offer insights for understanding the rich structural landscape of carbon, especially the 3D3C all-*sp*^2^ carbon networks.

## Methods

The calculations were carried out using the density functional theory as implemented in the Vienna *ab initio* simulation package (VASP)^24^. Both local density approximation (LDA) in the form of Ceperley-Alder^25^ and generalized gradient approximation (GGA) developed by Perdew, Burke and Ernzerhof (PBE)^26^ were adopted for the exchange-correlation potential. The all-electron projector augmented wave (PAW) method^27^ was adopted with the 2*s*^2^2*p*^2^ treated as valence electrons. A plane-wave basis set with an energy cutoff of 800 eV was used. Forces on the ions are calculated through the Hellmann-Feynman theorem allowing a full geometry optimization. Convergence criteria employed for both the electronic and the ionic relaxation were set to 10^−8^ eV and 0.01 eV/Å for energy and force, respectively. Electronic band structures are calculated using the hybrid functional (HSE06)^28^ under LDA and GGA. Phonon dispersion curves are calculated using the package MedeA^29^ with the forces calculated from VASP. To understand the kinetic stability of the new cR6 and cT8 carbon, the reaction barrier toward diamond and graphite are estimated using a generalized solid-state nudged elastic band method^9^,^37^,^38^ with the cell and atomic position optimization.

## Author Contributions

J.T.W. and C.F.C. designed the study and wrote the paper; J.T.W. and Y.K. carried out *ab initio* simulations; all authors discussed the results and contributed to the manuscript.

## Figures and Tables

**Table 1:** Calculated equilibrium structural parameters (space group, volume *V*, lattice parameters *a* and *c*, bond lengths *d_C–C_*), total energy *E_tot_*, bulk modulus *B*_0_, and electronic band gap *E_g_* for cT8 carbon, cR6 carbon, cH6 carbon, graphite, and diamond at zero pressure, compared to available experimental data^34^

Structure	Method	*V*_0_(Å^3^/atom)	a (Å)	c (Å)	*d_C–C_* (Å)	*E_tot_* (eV)	*B*_0_ (GPa)	*E_g_* (eV)
Diamond ( 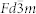 )	LDA	5.52	3.5336		1.530	−10.134	466	5.43
	PBE	5.70	3.5718		1.547	−9.097	433	5.27
	Exp^34^	5.67	3.5667		1.544		446	5.47
cT8 (*I*4_1_/*amd*)	LDA	8.22	5.8963	3.7843	1.343, 1.477	−9.639	296	2.38
	PBE	8.51	5.9307	3.8660	1.357, 1.491	−8.724	275	2.44
cR6 ( 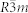 )	LDA	8.59	7.0544	3.5817	1.344, 1.479	−9.585	280	2.97
	PBE	8.88	7.1539	3.6023	1.353, 1.495	−8.678	262	2.93
cH6 (*P*6_2_22)	LDA	8.20	3.8867	3.7591	1.381, 1.459	−9.389	293	met
	PBE	8.49	3.9399	3.7879	1.391, 1.476	−8.467	272	met
Graphite (*P*6_3_/*mmc*)	LDA	8.55	2.4456	6.6046	1.412	−10.124	295	
	PBE	8.89	2.4600	6.7518	1.420	−9.214	274	
	Exp^34^	8.78	2.4600	6.704	1.420		286–319	

## References

[b1] Balaban A. T. Carbon and its nets. Computers Math. Applic. 17, 397–416 (1989).

[b2] Haley Michael M. On the road to carbyne. Nature Chem. 2, 912–913 (2010). 20966944 10.1038/nchem.884

[b3] Chalifoux W. A. & Tykwinski R. R. Synthesis of polyynes to model the *sp*-carbon allotrope carbyne. Nature Chem. 2, 967–971 (2010). 20966954 10.1038/nchem.828

[b4] Kroto H. W., Heath J. R., Obrien S. C., Curl R. F. & Smaliey R. E. C_60_: Buckminsterfullerene. Nature (London) 318, 162–163 (1985).

[b5] Iijima S. Helical microtubules of graphitic carbon. Nature (London) 354, 56–58 (1991).

[b6] Novoselov K. *et al.* Electric field effect in atomically thin carbon films. Science 306, 666–669 (2004). 15499015 10.1126/science.1102896

[b7] Mao W. L. *et al.* Bonding changes in compressed superhard graphite. Science 302, 425–427 (2003). 14564003 10.1126/science.1089713

[b8] Li Q. *et al.* Superhard monoclinic polymorph of carbon. Phys. Rev. Lett. 102, 175506 (2009). 19518796 10.1103/PhysRevLett.102.175506

[b9] Wang J. T., Chen C. F. & Kawazoe Y. Low-temperature phase transformation from graphite to *sp*^3^ orthorhombic carbon. Phys. Rev. Lett. 106, 075501 (2011). 21405524 10.1103/PhysRevLett.106.075501

[b10] Wang J. T., Chen C. F. & Kawazoe Y. Mechanism for direct conversion of graphite to diamond. Phys. Rev. B 84, 012102 (2011).

[b11] Wang J. T., Chen C. F. & Kawazoe Y. Orthorhombic carbon allotrope of compressed graphite: Ab initio calculations. Phys. Rev. B 85, 033410 (2012).

[b12] Wang J. T., Chen C. F. & Kawazoe Y. Phase conversion from graphite toward a simple monoclinic *sp*^3^-carbon allotrope. J. Chem. Phys. 137, 024502 (2012). 22803542 10.1063/1.4732538

[b13] Amsler M. *et al.* Crystal structure of cold compressed graphite. Phys. Rev. Lett. 108, 065501 (2012). 22401083 10.1103/PhysRevLett.108.065501

[b14] Li J. F. & Zhang R. Q. New superhard carbon allotropes based on C_20_ fullerene. Carbon 63, 571–573 (2013).

[b15] Gibson J., Holohan M. & Riley H. L. Amorphous carbon. J. Chem. Soc. 456–461 (1946). 20280686

[b16] O'Keeffe M., Adams G. B. & Sankey O. F. Predicted new low energy forms of carbon. Phys. Rev. Lett. 68, 2325–2328 (1992). 10045366 10.1103/PhysRevLett.68.2325

[b17] Wang J. T., Chen C. F. & Kawazoe Y. New cubic carbon phase via graphitic sheet rumpling. Phys. Rev. B 85, 214104 (2012).

[b18] Rajca A., Safronov A., Rajca S. & Shoemaker R. Double helical octaphenylene. Angew. Chem. Int. Ed. 36, 488–491 (1997).

[b19] Rignanese G.-M. & Charlier J.-C. Hypothetical three-dimensional all-*sp*^2^ carbon phase. Phys. Rev. B 78, 125415 (2008).

[b20] Tamor M. A. & Hass K. C. Hypothetical superhard carbon metal. J. Mater. Res. 5, 2273–2276 (1990).

[b21] Hoffmann R., Hughbanks T., Kertesz M. & Bird P. H. A hypothetical metallic allotrope of carbon. J. Am. Chem. Soc. 105, 4831–4832 (1983).

[b22] Itoh M., Kotani M., Naito H., Sunada T., Kawazoe Y. & Adschiri T. New metallic carbon crystal. Phys. Rev. Lett. 102, 055703 (2009). 19257523 10.1103/PhysRevLett.102.055703

[b23] Yao Y., Tse J. S., Sun J., Klug D. D., Martonak R. & Iitaka T. Comment on new metallic carbon crystal. Phys. Rev. Lett. 102, 229601 (2009). 19658907 10.1103/PhysRevLett.102.229601

[b24] Kresse G. & Furthmüller J. Efficient iterative schemes for *ab initio* total-energy calculations using a plane-wave basis set. Phys. Rev. B 54, 11169–11186 (1996). 10.1103/physrevb.54.11169 9984901

[b25] Perdew J. P. & Zunger A. Self-interaction correction to density-functional approximations for many-electron systems. Phys. Rev. B 23, 5048–5079 (1981).

[b26] Perdew J. P., Burke K. & Ernzerhof M. Generalized gradient approximation made simple. Phys. Rev. Lett. 77, 3865–3868 (1996). 10062328 10.1103/PhysRevLett.77.3865

[b27] Blöchl P. E. Projector augmented-wave method. Phys. Rev. B 50, 17953–17979 (1994). 10.1103/physrevb.50.17953 9976227

[b28] Krukau A. V., Vydrov O. A., Izmaylov A. F. & Scuseria G. E. Influence of the exchange screening parameter on the performance of screened hybrid functionals. J. Chem. Phys. 125, 224106 (2006). 17176133 10.1063/1.2404663

[b29] Parlinski K., Li Z. Q. & Kawazoe Y. First-principles determination of the soft mode in cubic ZrO_2_. Phys. Rev. Lett. 78, 4063–4066 (1997).

[b30] Choy T. P. & Kim Y. B. Classification of quantum phases for the star-lattice antiferromagnet via a projective symmetry group analysis. Phys. Rev. B 80, 064404 (2009).

[b31] Wang X. Q., Li H. D. & Wang J. T. Structural stabilities and electronic properties of planar C_4_ carbon sheet and nanoribbons. Phys. Chem. Chem. Phys. 14, 11107–11111 (2012). 22763793 10.1039/c2cp41464c

[b32] Batten S. R. & Robson R. Interpenetrating nets: ordered, periodic entanglement. Angew. Chem. Int. Ed. 37, 1460–1494 (1998). 10.1002/(SICI)1521-3773(19980619)37:11<1460::AID-ANIE1460>3.0.CO;2-Z 29710936

[b33] Evers J. Transformation of three-dimensional three-connected silicon nets in SrSi_2_. J. Solid State Chem. 24, 199–207 (1978).

[b34] Occelli F., Loubeyre P. & Letoullec R. Properties of diamond under hydrostatic pressures up to 140 GPa. Nature Mater. 2, 151–154 (2003). 12612670 10.1038/nmat831

[b35] Maultzsch J., Reich S., Thomsen C., Requardt H. & Ordejon P. Phonon dispersion in graphite. Phys. Rev. Lett. 92, 075501 (2004). 14995866 10.1103/PhysRevLett.92.075501

[b36] Nepal A., Singh G. P., Flanders B. N. & Sorensen C. M. One-step synthesis of graphene via catalyst-free gas-phase hydrocarbon detonation. Nanotechnology 24, 245602 (2013). 23690093 10.1088/0957-4484/24/24/245602

[b37] Sheppard D., Xiao P., Chemelewski W., Johnson D. D. & Henkelman G. A generalized solid-state nudged elastic band method. J. Chem. Phys. 136, 074103 (2012). 22360232 10.1063/1.3684549

[b38] Wang J. T., Chen C. F., Mizuseki H. & Kawazoe Y. Kinetic origin of divergent decompression pathways in silicon and germanium. Phys. Rev. Lett. 110, 165503 (2013). 23679617 10.1103/PhysRevLett.110.165503

